# Case Report: Atypical post-COVID Cogan's syndrome

**DOI:** 10.12688/f1000research.155250.1

**Published:** 2024-09-27

**Authors:** Sameh Mezri, Chaima Zitouni, Wafa Sleimi, Mayssa Bouzidi, Sayhi Sameh

**Affiliations:** 1 University of Tunis El-Manar, Tunis, Tunisia; 2 ENT Department, Military Hospital of Tunis, Montfleury, Tunis, 1008, Tunisia; 3 Department of Internal Medicine, Military Hospital of Tunis, Montfleury, Tunis, 1008, Tunisia

**Keywords:** Cogan Syndrome, Hearing Loss, Meningitis, COVID-19, Case report

## Abstract

**Background:**

Cogan’s syndrome is a rare autoimmune disorder characterized by ocular inflammation, vestibulocochlear dysfunction, and systemic vasculitis.

**Case Presentation:**

We report a 28-year-old female who experienced decreased visual acuity and ocular redness one month after a COVID-19 infection, with ophthalmological signs linked to keratitis, uveitis and retinal vascularitis. Two weeks later, she developed vertigo, tinnitus, and sudden hearing loss, leading to a diagnosis of Cogan’s disease. The patient received corticosteroid therapy, resulting in regression of ophthalmological signs, but progressed to complete deafness. One month later, she presented with lymphocytic meningitis and high intracranial pressure, which improved under treatment. The patient later received cochlear implants.

**Objective:**

This case report aims to highlight an atypical presentation of Cogan’s syndrome with neurological involvement following a COVID-19 infection. This case contributes to the limited literature on such presentations.

**Conclusion:**

Our case is one of only two reported instances of Cogan’s syndrome presenting with neurological signs post-COVID-19 infection, underscoring the rarity and complexity of this condition.

## Introduction

Cogan’s syndrome (CS) is a rare autoimmune systemic vasculitis characterized by ocular inflammation, vestibulocochlear dysfunction, and systemic vasculitis. ^1^ It was initially described in 1934 by Morgan and Baumgartner and later by David G. Cogan in 1945 as an association between interstitial keratitis and auditory symptoms. ^2^ Approximately 450 cases have been documented to date. ^1^

We present a case of atypical Cogan’s syndrome, distinguished by its onset following a COVID-19 infection and unusual features including ophthalmic conditions beyond keratitis and the development of neurological symptoms.

To our knowledge, this is the second reported instance of post-COVID Cogan’s syndrome in the literature. ^3^

The purpose of this article was to explore through our case, the pathophysiology and clinical features of Cogan’s syndrome as well as to review its treatment options, prognosis, and progression.

## Case report

We present the case of a 28-year-old woman with a recent history of COVID-19 infection. One month after the infection, she developed ophthalmic abnormalities, including eye redness, blurred vision, and decreased visual acuity. These symptoms were related to bilateral non-granulomatous anterior uveitis, bilateral interstitial keratitis, retinal vasculitis, and bilateral stage 2 papilledema. She was treated with local and systemic corticosteroids, leading to improvement. Serologies for syphilis, brucellosis, Human Immunodeficiency Virus (HIV), hepatitis B virus, hepatitis C virus, rheumatoid factor, antibodies against SSA, SSB, antinuclear, anticardiolipin, and antineutrophil, and tuberculin intradermal reaction (IDR) were performed to investigate potential autoimmune or infectious origins, all of which were negative.

Two weeks after the onset of ocular symptoms, the patient developed vertigo, bilateral tinnitus, and sudden hearing loss. After excluding neurological causes with normal magnetic resonance imaging (MRI) results, she was referred to our department. The vestibular examination revealed signs suggestive of a peripheral origin. Initial pure-tone audiometry revealed moderate sensorineural hearing loss (45 dB) in both ears. Laboratory tests showed a mild inflammatory syndrome (C-reactive protein [CRP] = 15 mg/L, platelet count [PLT] = 489 × 10 ^9^/L). The patient was treated with Methylprednisolone (1 mg/kg per day) and Acetylleucine (20 mg three times daily).

**Figure 1.**
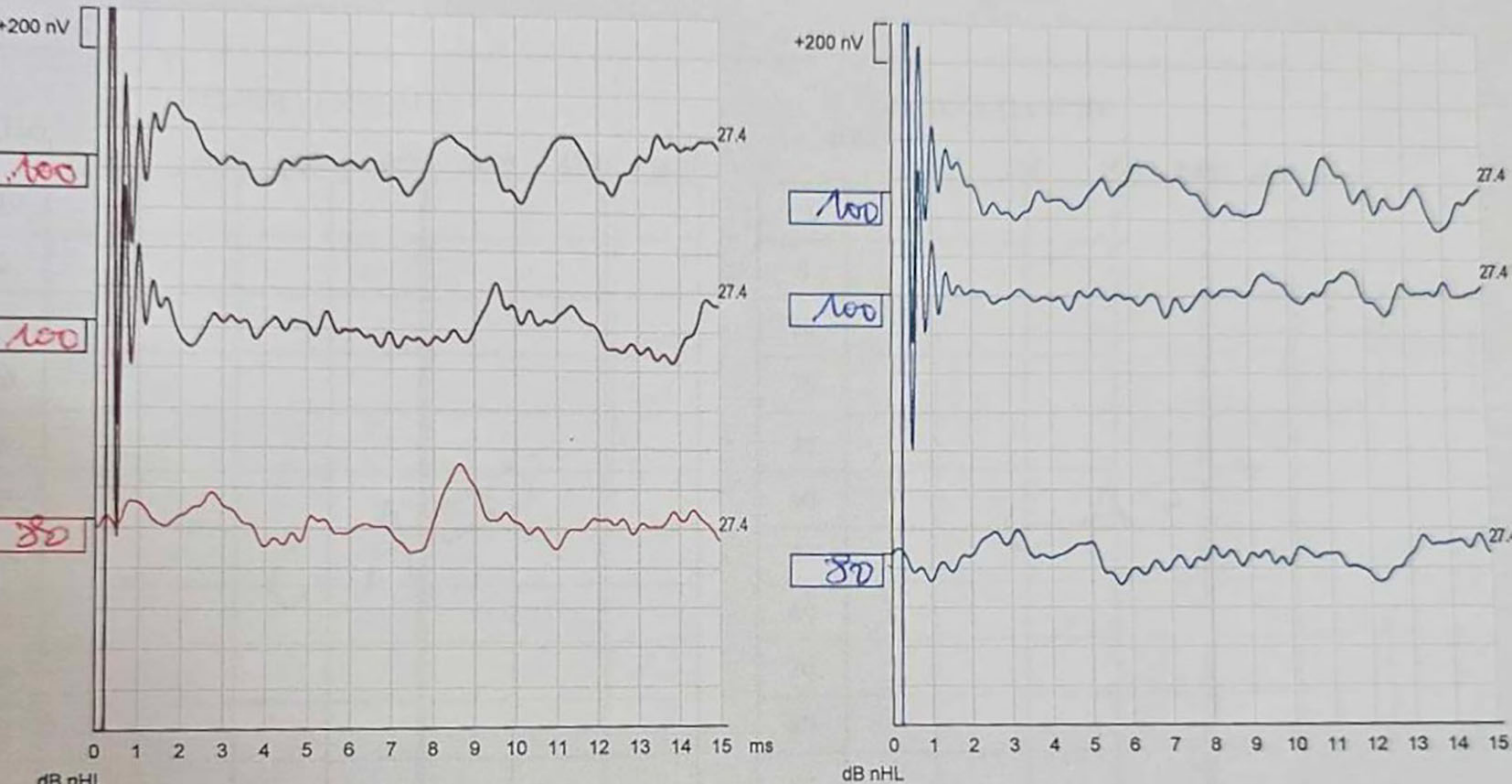
Auditory Brainstem Response (ABR). The patient's ABR shows the absence of Wave V at 100 dB for both ears. The right ear is depicted in red and the left ear in blue.

Despite treatment, audiometric control showed worsening hearing loss (50 dB). The patient underwent 10 sessions of hyperbaric oxygen therapy, but subsequent audiograms revealed complete deafness, confirmed by auditory brainstem response (ABR) ( Figure 1). Given the rapid progression to deafness, Cogan’s syndrome was diagnosed.

One month later, the patient developed headaches, dizziness, and vomiting, leading to readmission. Cerebral spinal fluid analysis showed lymphocytosis (33 white blood cells). We conducted cultures, polymerase chain reaction (PCR) for specific viruses, serological tests, and auto-immune markers, and they all came back negative. Computed tomography (CT) angiography ruled out cerebral thrombosis. Ophthalmological examination revealed episcleritis and bilateral stage 2 papilledema. Meningitis and high intracranial pressure (HTIC) were manifestations of Cogan’s disease. The patient was treated with corticosteroids and acetazolamide, leading to a good outcome.

Later on, she received cochlear implants in both ears. Two years after the diagnosis, the patient was doing well.

## Discussion

Cogan’s syndrome (CS) is a systemic inflammatory disease characterized by vasculitis affecting both small and large vessels. ^4^ It is characterized by interstitial keratitis and sensorineural hearing loss, often associated with systemic vasculitis. ^4^ While the exact pathogenesis remains unclear, some reports suggest an inflammatory autoimmune mechanism or a post-infectious etiology. ^5^ There have been instances of CS following infections, typically occurring within 7 to 10 days of upper respiratory infections in 32% to 65% of cases. ^5^ The mechanism might involve molecular mimicry triggered by viral infections. ^5^

Since the onset of the COVID-19 pandemic, several reports have emerged regarding autoimmune manifestations and sequelae of this infection. ^6^ Notably, only one case of CS following a COVID-19 infection has been documented in the literature. ^3^ Our case represents the second reported instance.

This condition typically affects young Caucasian adults without gender or hereditary predispositions. ^2^ Initial symptoms often involve either ocular disturbances (40%) or auditory-vestibular disturbances (40%), with both the eye and ear being simultaneously affected in about 16% of cases. ^1^^,^^5^

The classic ophthalmic presentation of CS is characterized by ocular issues such as keratitis, which may be accompanied by uveitis or conjunctivitis. The classic otolaryngological presentation includes cochlear and vestibular symptoms similar to those of Meniere’s disease. Hearing loss in CS typically affects both ears. Typically, ocular and auditory-vestibular symptoms develop within two years of each other. ^4^^,^^3^

In CS, vestibulocochlear dysfunction is associated with inflammation in the cochlea, neuronal loss in the auditory system, endolymphatic hydrops, degeneration of the Organ of Corti, new bone formation, and atrophy of the vestibulocochlear nerve. ^7^

Atypical CS is marked by the absence of interstitial keratitis or a time gap of more than two years between cochleovestibular and ocular manifestations. ^5^^,^^8^ Unusual ocular symptoms in atypical cases may include retinal vasculitis, papillitis, central retinal occlusion, scleritis, episcleritis, and uveitis. ^3^^,^^6^ In contrast to typical forms, atypical CS is often associated with a broader range of systemic manifestations, such as fever, weight loss, respiratory issues, gastrointestinal bleeding, lymphadenopathy, splenomegaly, musculoskeletal symptoms, cardiovascular problems, and neurological signs. ^5^^,^^8^ Meningitis has also been reported as a neurological manifestation of CS. ^1^ Our case illustrates atypical CS with unusual ocular and neurological involvement.

Diagnosing CS relies on its clinical manifestations and the exclusion of other conditions. ^1^ While there are no specific diagnostic laboratory or radiologic tests, anti-Hsp70 antibodies have been suggested as a potential serological marker for typical CS. ^9^ Vestibular testing (e.g., caloric testing, electronystagmography) and audiometry often reveal abnormalities, but no specific diagnostic pattern confirms the diagnosis. ^1^ Hearing loss typically involves high frequencies, and autoantibodies and serologic markers of inflammation may be present. ^1^

Currently, there are no precise guidelines for diagnosing this syndrome, and diagnosis is often delayed or missed due to the rarity and non-specific nature of the symptoms. ^1^^,^^4^^,^^5^ Management strategies are tailored to the severity and organ involvement, focusing on preventing irreversible damage. Treatments include antibiotics, vaccines, convalescent plasma, low-salt diets, vitamins, and immunosuppressive therapies. ^10^ Corticosteroids are the first-line treatment for managing ocular, vascular, and visceral symptoms, although their effectiveness on audio-vestibular symptoms is limited. ^2^ Early implantation, ideally within 8 weeks of hearing loss onset, can help mitigate complications such as cochlear fibrosis. ^11^

In CS, ophthalmic disease often fluctuates with periods of remission. ^2^ Although ocular involvement generally has a better prognosis, it can lead to long-term complications such as cataracts from corticosteroid use. ^2^ Conversely, auditory involvement frequently results in complete and irreversible bilateral sensorineural deafness. ^2^

In our case, despite the resolution of ophthalmic and neurological symptoms, the patient did not respond to treatment for her hearing loss, which progressed to profound deafness. As a result, she underwent cochlear implantation. This progression aligns with findings reported in the literature.

CS has a 10% mortality rate, largely due to complications arising from vasculitis, including stroke, gastrointestinal bleeding, cardiac problems, and systemic vasculitis. ^12^

## Conclusion

CS is difficult to assess because it overlaps with many other diagnoses due to the variety of symptoms. Diagnosis is generally delayed until the main criteria are met and other diagnoses are excluded. Despite these difficulties, early diagnosis is essential to initiate appropriate treatment, although treatment appears to be more effective for ocular symptoms than for hearing loss, which often progresses to profound deafness. Since it is a multi-organ condition, regular follow-up is essential. Finally, the literature data is limited; therefore, further studies are needed to better understand its pathophysiology and treatment.

## Consent to publish

Written informed consent was obtained from our patient for anonymously published cases. The local ethical committee of the military hospital of Tunis, Tunisia authorized the publication of the case.

## Data Availability

No data are associated with this article.
