# Case Report: Atypical post-COVID Cogan's syndrome

**DOI:** 10.12688/f1000research.155250.2

**Published:** 2024-12-20

**Authors:** Sameh Mezri, Chaima Zitouni, Wafa Sleimi, Mayssa Bouzidi, Sayhi Sameh

**Affiliations:** 1 University of Tunis El-Manar, Tunis, Tunisia; 2 ENT Department, Military Hospital of Tunis, Montfleury, Tunis, 1008, Tunisia; 3 Department of Internal Medicine, Military Hospital of Tunis, Montfleury, Tunis, 1008, Tunisia

**Keywords:** Cogan Syndrome, Hearing Loss, Meningitis, COVID-19, Case report

## Abstract

**Background:**

Cogan’s syndrome is a rare autoimmune disorder characterized by ocular inflammation, vestibulocochlear dysfunction, and systemic vasculitis.

**Case Presentation:**

We report a 28-year-old female who experienced decreased visual acuity and ocular redness one month after a COVID-19 infection, with ophthalmological signs linked to keratitis, uveitis and retinal vasculitis. Two weeks later, she developed vertigo, tinnitus, and sudden hearing loss, leading to a diagnosis of Cogan’s disease. The patient received corticosteroid therapy, resulting in regression of ophthalmological signs, but progressed to complete deafness. One month later, she presented with lymphocytic meningitis and high intracranial pressure, which improved under treatment. The patient later received cochlear implants.

**Objective:**

This case report aims to highlight an atypical presentation of Cogan’s syndrome with neurological involvement following a COVID-19 infection. This case contributes to the limited literature on such presentations.

**Conclusion:**

Our case is one of only two reported instances of Cogan’s syndrome presenting with neurological signs post-COVID-19 infection, underscoring the rarity and complexity of this condition.

## Introduction

Cogan’s syndrome (CS) is a rare autoimmune systemic vasculitis characterized by ocular inflammation, vestibulocochlear dysfunction, and systemic vasculitis. ^1^ It was initially described in 1934 by Morgan and Baumgartner and later by David G. Cogan in 1945 as an association between interstitial keratitis and auditory symptoms. ^2^ Approximately 450 cases have been documented to date. ^1^

We present a case of atypical Cogan’s syndrome, distinguished by its onset following a COVID-19 infection and unusual features including ophthalmic conditions beyond keratitis and the development of neurological symptoms.

To our knowledge, this is the second reported instance of post-COVID Cogan’s syndrome in the literature. ^3^

The purpose of this article was to explore through our case, the pathophysiology and clinical features of Cogan’s syndrome as well as to review its treatment options, prognosis, and progression.

## Case report

We present the case of a 28-year-old woman with a recent history of COVID-19 infection. One month after the infection, she developed ophthalmic abnormalities, including eye redness, blurred vision, and decreased visual acuity. She consulted 3 days after the onset of symptoms and reported no associated arthralgia, fever, or weight loss.

Ophthalmological examination revealed red eyes, bilateral non-granulomatous anterior uveitis, bilateral interstitial keratitis, retinal vasculitis, and bilateral stage 2 papilledema. The rest of the examination was unremarkable.

Initially, we conducted serologies for HIV, syphilis, brucellosis, hepatitis B and C, and tuberculin intradermal reaction (IDR) to exclude common infectious causes. After these tests returned negative, we proceeded with autoimmune screening, including antinuclear antibodies (ANA), anti-DNA antibodies, rheumatoid factor (RF), anti-SSA/SSB antibodies, anticardiolipin antibodies, and antineutrophil cytoplasmic antibodies (ANCA), to investigate potential autoimmune disease.

The patient was treated with local and systemic corticosteroids (Methylprednisolone (1 mg/kg per day)), leading to improvement.

Two weeks after the onset of ocular symptoms, the patient developed vertigo, bilateral tinnitus, and sudden hearing loss. After excluding neurological causes with normal neurological exam and magnetic resonance imaging (MRI) results, she was referred to our department. The vestibular examination revealed signs suggestive of a peripheral origin, including horizontal-rotary nystagmus, positive Head Impulse Test (HIT), and balance instability in static and dynamic tests.

Initial pure-tone audiometry revealed moderate sensorineural hearing loss (45 dB) in both ears. Laboratory tests showed a mild inflammatory syndrome (C-reactive protein [CRP] = 15 mg/L, platelet count [PLT] = 489 × 10 ^9^/L).

The corticosteroid treatment was maintained at the same dose (1 mg/kg/day). The patient also recieved Acetylleucine (20 mg three times daily) for 4 days.

**Figure 1.**
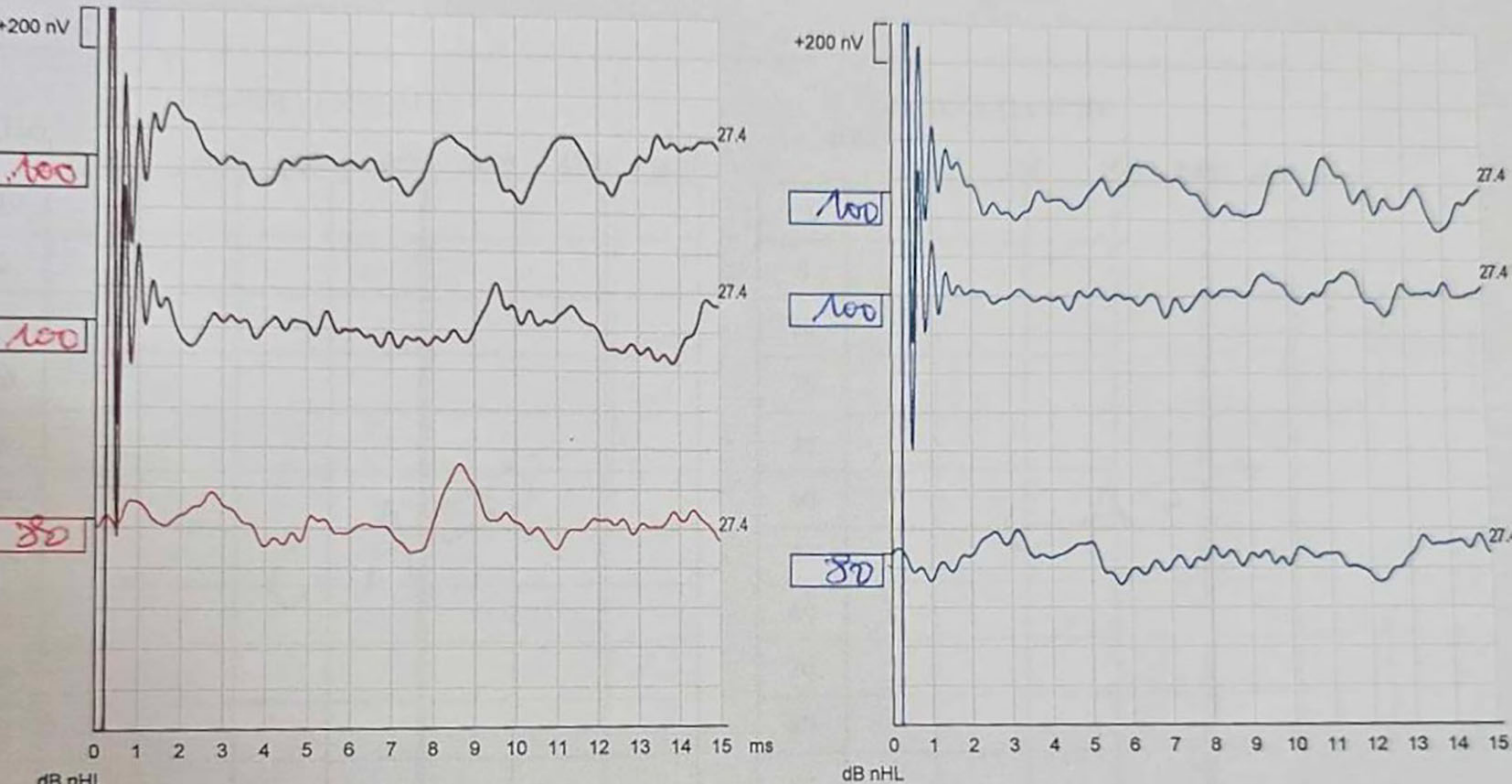
Auditory Brainstem Response (ABR). The patient's ABR shows the absence of Wave V at 100 dB for both ears. The right ear is depicted in red and the left ear in blue.

Despite treatment, audiometric control showed worsening hearing loss (50 dB) after 7 days. The patient underwent 10 sessions of hyperbaric oxygen therapy, but subsequent audiograms revealed complete deafness 12 days after treatment, confirmed by auditory brainstem response (ABR) ( Figure 1). Given the rapid progression to deafness, Cogan’s syndrome was diagnosed.

One month later, the patient developed headaches, dizziness, and vomiting, leading to readmission. Cerebral spinal fluid analysis showed lymphocytosis (33 white blood cells). We conducted cultures, polymerase chain reaction (PCR) for specific viruses, serological tests, and auto-immune markers, and they all came back negative. Computed tomography (CT) angiography ruled out cerebral thrombosis. Ophthalmological examination revealed episcleritis and bilateral stage 2 papilledema. Meningitis and high intracranial pressure (HTIC) were manifestations of Cogan’s disease. The patient was treated with corticosteroids and acetazolamide, leading to a good outcome.

Later on, she received cochlear implants in both ears. Two years after the diagnosis, the patient was doing well, with no ophthalmological signs or other symptoms. She was regularly followed by neurology, ophthalmology, otorhinolaryngology (ENT), and internal medicine specialists.

## Discussion

Cogan’s syndrome (CS) is a systemic inflammatory disease characterized by vasculitis affecting both small and large vessels. ^4^ It is characterized by interstitial keratitis and sensorineural hearing loss, often associated with systemic vasculitis. ^4^ While the exact pathogenesis remains unclear, some reports suggest an inflammatory autoimmune mechanism or a post-infectious etiology. ^5^ There have been instances of CS following infections, typically occurring within 7 to 10 days of upper respiratory infections in 32% to 65% of cases. ^5^

Since the onset of the COVID-19 pandemic, several reports have emerged regarding autoimmune manifestations and sequelae of this infection. ^6^ Notably, only one case of CS following a COVID-19 infection has been documented in the literature. ^3^ Our case represents the second reported instance. The mechanism might involve molecular mimicry triggered by viral infections leading to autoimmune diseases. ^5^ This immune response can damage to inner ear structures like the cochlear epithelium and vestibulocochlear nerve. The virus’s neuro-invasive potential could also play a role in affecting auditory pathways. ^7^

CS typically affects young Caucasian adults without gender or hereditary predispositions. ^2^ Initial symptoms often involve either ocular disturbances (40%) or auditory-vestibular disturbances (40%), with both the eye and ear being simultaneously affected in about 16% of cases. ^1^^,^^5^

The classic ophthalmic presentation of CS is characterized by ocular issues such as keratitis, which may be accompanied by uveitis or conjunctivitis. The classic otolaryngological presentation includes cochlear and vestibular symptoms similar to those of Meniere’s disease. Hearing loss in CS typically affects both ears. Typically, ocular and auditory-vestibular symptoms develop within two years of each other. ^4^^,^^3^

In CS, vestibulocochlear dysfunction is associated with inflammation in the cochlea, neuronal loss in the auditory system, endolymphatic hydrops, degeneration of the Organ of Corti, new bone formation, and atrophy of the vestibulocochlear nerve. ^8^

Atypical CS is marked by the absence of interstitial keratitis or a time gap of more than two years between cochleovestibular and ocular manifestations. ^5^^,^^9^ Unusual ocular symptoms in atypical cases may include retinal vasculitis, papillitis, central retinal occlusion, scleritis, episcleritis, and uveitis. ^3^^,^^6^ In contrast to typical forms, atypical CS is often associated with a broader range of systemic manifestations, such as fever, weight loss, respiratory issues, gastrointestinal bleeding, lymphadenopathy, splenomegaly, musculoskeletal symptoms, cardiovascular problems, and neurological signs. ^5^^,^^9^ Meningitis has also been reported as a neurological manifestation of CS. ^1^^,^^10^ Our case illustrates atypical CS with unusual ocular and neurological involvement.

Diagnosing CS relies on its clinical manifestations and the exclusion of other conditions. ^1^ While there are no specific diagnostic laboratory or radiologic tests, anti-Hsp70 antibodies have been suggested as a potential serological marker for typical CS. ^11^ Vestibular testing (e.g., caloric testing, electronystagmography) and audiometry often reveal abnormalities, but no specific diagnostic pattern confirms the diagnosis. ^1^ Hearing loss typically involves high frequencies, and autoantibodies and serologic markers of inflammation may be present. ^1^

Currently, there are no precise guidelines for diagnosing this syndrome, and diagnosis is often delayed or missed due to the rarity and non-specific nature of the symptoms. ^1^^,^^4^^,^^5^

While the differential diagnosis of concurrent ocular and audiovestibular symptoms includes conditions such as congenital syphilis and autoimmune diseases like Susac syndrome or Vogt-Koyanagi-Harada syndrome; ^12^ autoimmune diseases commonly associated with isolated sensorineural hearing loss include systemic lupus erythematosus, rheumatoid arthritis, and vitiligo. ^13^ This is why it is essential to rule out certain infectious and autoimmune conditions by performing serological tests and a comprehensive autoimmune workup before confirming the diagnosis of CS.

Radiological evaluation, particularly through MRI, is also important in distinguishing autoimmune inner ear disease from other causes like infectious or vascular etiologies. Post-contrast T1-weighted imaging reveals disruptions in the blood-labyrinth barrier with enhancement patterns indicative of inflammation, while post-contrast FLAIR provides superior sensitivity for detecting early gadolinium leakage into inner ear fluids, often bilateral in autoimmune inner ear disease. In contrast, vascular etiologies such as labyrinthine artery infarction demonstrate restricted diffusion on diffusion-weighted imaging without enhancement, and infectious labyrinthitis typically shows unilateral enhancement accompanied by middle ear effusions. ^14^

Management strategies are tailored to the severity and organ involvement, focusing on preventing irreversible damage.

Corticosteroids remain the first-line treatment for CS. They help manage ocular symptoms more effectively than audio-vestibular issues, and early intervention with high-dose steroids is crucial, with improvements often seen within 2–3 weeks. For cases resistant to steroids, other immunosuppressive agents, including cyclophosphamide, methotrexate, azathioprine, and cyclosporine A, are considered, though their effectiveness varies. Biological therapies, such as TNFα inhibitors and rituximab, offer promising alternatives, especially in refractory cases, though evidence is still limited. ^15^

To prevent complications from immunosuppression in the treatment of CS, prophylactic measures should include routine monitoring for infections, managing vaccination schedules (e.g., live vaccines should be avoided during immunosuppressive therapy), and regular screening for common side effects of immunosuppressants such as bone loss, liver toxicity, and gastrointestinal issues. ^15^ Additionally, strategies like calcium and vitamin D supplementation, along with proton pump inhibitors for gastric protection, should be considered to minimize long-term adverse effects from corticosteroids and other immunosuppressive drugs. ^15^

Early implantation, ideally within 8 weeks of hearing loss onset, can help mitigate complications such as cochlear fibrosis. ^16^

In CS, ophthalmic disease often fluctuates with periods of remission. ^2^ Although ocular involvement generally has a better prognosis, it can lead to long-term complications such as cataracts from corticosteroid use. ^2^ Conversely, auditory involvement frequently results in complete and irreversible bilateral sensorineural deafness. ^2^

In our case, despite the resolution of ophthalmic and neurological symptoms, the patient did not respond to treatment for her hearing loss, which progressed to profound deafness. As a result, she underwent cochlear implantation. This progression aligns with findings reported in the literature.

CS has a 10% mortality rate, largely due to complications arising from vasculitis, including stroke, gastrointestinal bleeding, cardiac problems, and systemic vasculitis. ^17^

### Strengths and limitations

This case report highlights the rare, atypical presentation of CS following a COVID-19 infection, contributing valuable insights to the limited literature on post-infectious autoimmune disorders. However, the study’s limitations include its retrospective design, lack of long-term immunological follow-up, and the fact that it represents a single case, which may limit generalizability.

## Conclusion

CS is difficult to assess because it overlaps with many other diagnoses due to the variety of symptoms. Diagnosis is generally delayed until the main criteria are met and other diagnoses are excluded. Despite these difficulties, early diagnosis is essential to initiate appropriate treatment, although treatment appears to be more effective for ocular symptoms than for hearing loss, which often progresses to profound deafness. Since it is a multi-organ condition, regular follow-up is essential. Finally, the literature data is limited; therefore, further studies are needed to better understand its pathophysiology particularly post-COVID autoimmune conditions, CS treatment, as well as optimal timing and long-term outcomes of cochlear implantation in autoimmune-related hearing loss.

## Consent to publish

Written informed consent was obtained from our patient for anonymously published cases. The local ethical committee of the military hospital of Tunis, Tunisia authorized the publication of the case.

## Data Availability

No data are associated with this article.
