# Supplementary material for: Case Report: Atypical post-COVID Cogan's syndrome
Source: F1000Res. 2024 Dec 20;13:1104. Originally published 2024 Sep 27. [Version 2] doi: 10.12688/f1000research.155250.2 (PMC11809643; doi:10.12688/f1000research.155250.2)
Supplement: Peer review history

# Reviewer report: Reviewer response for version 2

**DOI:** 10.5256/f1000research.175760.r351350

**Published:** 2025-01-06

**Authors:** Andrea Frosolin

**Affiliations:** 1 University of Siena, Siena, Italy

The authors have substantially improved the manuscript which is now suitable for indexing.

Are enough details provided of any physical examination and diagnostic tests, treatment given and outcomes?

Partly

Is the case presented with sufficient detail to be useful for other practitioners?

Partly

Is sufficient discussion included of the importance of the findings and their relevance to future understanding of disease processes, diagnosis or treatment?

Partly

Is the background of the case’s history and progression described in sufficient detail?

Partly

Reviewer Expertise:

NA

I confirm that I have read this submission and believe that I have an appropriate level of expertise to confirm that it is of an acceptable scientific standard.

# Reviewer report: Reviewer response for version 1

**DOI:** 10.5256/f1000research.170398.r340628

**Published:** 2024-12-05

**Authors:** Andrea Frosolin

**Affiliations:** 1 University of Siena, Siena, Italy

Thank you for the opportunity to review the manuscript titled *“Case Report: Atypical Post-COVID Cogan's Syndrome”*. This case highlights an important and rare association of Cogan’s syndrome (CS) following COVID-19, emphasizing the evolving understanding of autoimmune and post-infectious complications. Below are detailed recommendations and suggestions for improvement.

 The manuscript would benefit from an expanded discussion of the differential diagnosis of audio-vestibular pathologies after COVID (Frosolini A, et al., 2022 [Ref 1]).

 Align the case report structure with the **CARE guidelines** to enhance clarity and comprehensiveness. This includes ensuring that all relevant patient history, diagnostic evaluations, and follow-up details are systematically presented.

 While several laboratory tests are reported, it would be useful to include additional markers such as complement levels, anti-dsDNA antibodies, or other autoimmune markers to strengthen the exclusion of alternative diagnoses, particularly systemic lupus erythematosus (SLE). The role of imaging could be further elaborated. Include details about the radiological evaluation process, and consider discussing its utility in identifying autoimmune-related inner ear involvement compared to infectious or vascular etiologies.

 While corticosteroid therapy was described, the discussion could benefit from exploring alternative or adjunctive treatments such as steroid-sparing immunosuppressive agents (e.g., methotrexate or azathioprine). Additionally, prophylactic measures to prevent complications from immunosuppression should be briefly discussed.

 Add a dedicated section summarizing the strengths (e.g., highlighting an underreported clinical entity) and limitations (e.g., single case, absence of long-term immunological follow-up) of the study. A short paragraph could highlight areas for future research, such as the need for studies exploring the pathophysiology of post-COVID autoimmune conditions or the timing and outcomes of cochlear implantation in autoimmune hearing loss.

 Possible typo: "vascularitis".

Are enough details provided of any physical examination and diagnostic tests, treatment given and outcomes?

Partly

Is the case presented with sufficient detail to be useful for other practitioners?

Partly

Is sufficient discussion included of the importance of the findings and their relevance to future understanding of disease processes, diagnosis or treatment?

Partly

Is the background of the case’s history and progression described in sufficient detail?

Partly

Reviewer Expertise:

audiology

I confirm that I have read this submission and believe that I have an appropriate level of expertise to confirm that it is of an acceptable scientific standard, however I have significant reservations, as outlined above.

## References

[1] : Sudden Sensorineural Hearing Loss in the COVID-19 Pandemic: A Systematic Review and Meta-Analysis. *Diagnostics (Basel)* .2022;12(12) : 10.3390/diagnostics12123139 10.3390/diagnostics12123139 36553146 PMC9777296

# Response

**Published:** 2024-12-11

**Authors:** Chaima zitouni

We sincerely thank Dr. Andrea Frosolin for the thoughtful review and valuable comments, and here are our detailed responses to the comments provided:

1. **The manuscript would benefit from an expanded discussion of the differential diagnosis of audio-vestibular pathologies after COVID (Frosolini A, et al., 2022 [Ref 1]).**

We have expanded the discussion on the differential diagnosis of audio-vestibular pathologies post-COVID, incorporating insights from Frosolini et al. (2022).

1. **Align the case report structure with the CARE guidelines to enhance clarity and comprehensiveness. This includes ensuring that all relevant patient history, diagnostic evaluations, and follow-up details are systematically presented.**

We have carefully detailed the case in accordance with the CARE guidelines to ensure clarity and comprehensiveness.

1. **While several laboratory tests are reported, it would be useful to include additional markers such as complement levels, anti-dsDNA antibodies, or other autoimmune markers to strengthen the exclusion of alternative diagnoses, particularly systemic lupus erythematosus (SLE). The role of imaging could be further elaborated. Include details about the radiological evaluation process, and consider discussing its utility in identifying autoimmune-related inner ear involvement compared to infectious or vascular etiologies.**

The anti-DNA antibody was tested (we have added this to the revised text), while the complement levels were not measured.

 We have expanded the differential diagnoses, particularly focusing on systemic lupus erythematosus and other autoimmune diseases.

 We have expanded the discussion to detail the radiological evaluation process, emphasizing the role of advanced MRI sequences in distinguishing autoimmune-related inner ear involvement from infectious or vascular etiologies based on characteristic patterns.

1. **While corticosteroid therapy was described, the discussion could benefit from exploring alternative or adjunctive treatments such as steroid-sparing immunosuppressive agents (e.g., methotrexate or azathioprine). Additionally, prophylactic measures to prevent complications from immunosuppression should be briefly discussed.**

In response to your suggestion, we have expanded the discussion to include steroid-sparing immunosuppressive agents such as methotrexate and azathioprine…

 We have added a discussion of the prophylactic measures to prevent complications arising from immunosuppressive treatments.

1. **Add a dedicated section summarizing the strengths (e.g., highlighting an underreported clinical entity) and limitations (e.g., single case, absence of long-term immunological follow-up) of the study. A short paragraph could highlight areas for future research, such as the need for studies exploring the pathophysiology of post-COVID autoimmune conditions or the timing and outcomes of cochlear implantation in autoimmune hearing loss.**

We have included a dedicated section in the discussion summarizing the strengths and limitations of our study.

 A short paragraph has been added to highlight areas for future research.

  

1. **Possible typo: "vascularitis".**

We have corrected the typo from "vascularitis" to "vasculitis" as per your suggestion.

# Reviewer report: Reviewer response for version 1

**DOI:** 10.5256/f1000research.170398.r330430

**Published:** 2024-11-06

**Authors:** Debashis Maikap

**Affiliations:** 1 Kalinga Institute of Industrial Technology (KIIT), Odisha, India

This case highlights a rare post-COVID-19 complication that progressed to Cogan’s syndrome, a unique autoimmune disorder affecting the eyes and ears. The syndrome commonly presents as non-syphilitic interstitial keratitis along with audio vestibular symptoms such as vertigo, tinnitus, and sensorineural hearing loss. Below are key points noted for improvement and additions

1.  Corrections were made for terms like "retinal vasculitis" rather than "vascularitis" to maintain accuracy in clinical descriptions.- ABSTRACT SECTION
2. Although the case notes a possible autoimmune response triggered by COVID-19, adding context on the immune response patterns in COVID-19 and their role in post-infectious autoimmune diseases like CS would enhance the discussion. The link between viral triggers and autoimmune conditions via mechanisms such as molecular mimicry can deepen understanding of disease etiology.
3. The use of high-dose corticosteroids presents infection risks, yet there was no mention of prophylactic measures, such as vaccinations or antibiotic prophylaxis, which are critical in managing immunosuppressed patients. Inclusion of these preventive strategies would provide a more comprehensive view of patient safety in long-term treatment.
4. Immunosuppressive agents such as cyclophosphamide, mycophenolate mofetil (MMF), or azathioprine, often utilized as steroid-sparing therapies, were not included in this case report as potential adjunctive treatments for achieving long-term systemic control. In atypical CS cases with progressive symptoms, early initiation of these therapies might have contributed to improved outcomes.
5. Complement levels and anti-double-stranded DNA (anti-dsDNA) antibodies, critical for ruling out lupus as an alternative diagnosis, were not mentioned.
6. References Update: The following reference has been added to support the discussion on atypical presentations of Cogan’s syndrome:

Maikap D, Pradhan A, Padhan P. A rare case of atypical Cogan's syndrome presenting as encephalitis. Mod Rheumatol Case Rep. 2022 Jun 24;6(2):305-308. doi: 10.1093/mrcr/rxab055. PMID: 34957524 (ref-1).

Are enough details provided of any physical examination and diagnostic tests, treatment given and outcomes?

Yes

Is the case presented with sufficient detail to be useful for other practitioners?

Yes

Is sufficient discussion included of the importance of the findings and their relevance to future understanding of disease processes, diagnosis or treatment?

Partly

Is the background of the case’s history and progression described in sufficient detail?

Yes

Reviewer Expertise:

Reactive arthritis, vasculitis, lupus

I confirm that I have read this submission and believe that I have an appropriate level of expertise to confirm that it is of an acceptable scientific standard.

## References

[1] : A rare case of atypical Cogan's syndrome presenting as encephalitis. *Mod Rheumatol Case Rep* .2022;6(2) : 10.1093/mrcr/rxab055 305-308 10.1093/mrcr/rxab055 34957524

# Response

**Published:** 2024-12-11

**Authors:** Chaima zitouni

We sincerely thank Dr. Debashis Maikap for the thoughtful review and valuable comments, and here are our detailed responses to the comments provided:

1. Corrections were made for terms like "retinal vasculitis" rather than "vascularitis" to maintain accuracy in clinical descriptions

Response: We have corrected the typo from "vascularitis" to "vasculitis" as per your suggestion.

  

1. Although the case notes a possible autoimmune response triggered by COVID-19, adding context on the immune response patterns in COVID-19 and their role in post-infectious autoimmune diseases like CS would enhance the discussion. The link between viral triggers and autoimmune conditions via mechanisms such as molecular mimicry can deepen understanding of disease etiology.

Response: We have included in the discussion an analysis of the potential mechanisms linking autoimmune pathologies to COVID-19.

  

1. The use of high-dose corticosteroids presents infection risks, yet there was no mention of prophylactic measures, such as vaccinations or antibiotic prophylaxis, which are critical in managing immunosuppressed patients. Inclusion of these preventive strategies would provide a more comprehensive view of patient safety in long-term treatment.

Response: We have added a discussion of the prophylactic measures to prevent complications arising from immunosuppressive treatments.

  

1. Immunosuppressive agents such as cyclophosphamide, mycophenolate mofetil (MMF), or azathioprine, often utilized as steroid-sparing therapies, were not included in this case report as potential adjunctive treatments for achieving long-term systemic control. In atypical CS cases with progressive symptoms, early initiation of these therapies might have contributed to improved outcomes.

Response: In response to your suggestion, we have expanded the discussion to include steroid-sparing immunosuppressive agents such as methotrexate and azathioprine…

  

1. Complement levels and anti-double-stranded DNA (anti-dsDNA) antibodies, critical for ruling out lupus as an alternative diagnosis, were not mentioned.

Response: The anti-DNA antibody was tested (we have added this to the revised text), while the complement levels were not measured.

  

1. References Update: The following reference has been added to support the discussion on atypical presentations of Cogan’s syndrome: Maikap D, Pradhan A, Padhan P. A rare case of atypical Cogan's syndrome presenting as encephalitis. Mod Rheumatol Case Rep. 2022 Jun 24;6(2):305-308. doi: 10.1093/mrcr/rxab055. PMID: 34957524 (ref-1).

Response: We have added the requested reference to the manuscript and integrated it into the relevant section of the discussion.
